# Non-adjustable surgery for acute acquired comitant esotropia under general anesthesia

**DOI:** 10.1186/s12886-022-02634-1

**Published:** 2022-11-01

**Authors:** Soo Hyun Lim, Yoon Gon Lee, Ungsoo Samuel Kim

**Affiliations:** 1 Department of Ophthalmology, Seoul National University Hospital, Seoul, Republic of Korea; 2 Kim’s Eye Hospital, Seoul, Republic of Korea; 3 Department of Ophthalmology, Gwangmyeong Hospital, Chung-Ang University, 110, Deokan-ro, 14353 Gwangmyeong-si, Gyeonggi-do Republic of Korea; 4 Gwangmyeong Hospital, Chung-Ang University, 110, Deokan-ro, 14353 Gwangmyeong-si, Gyeonggi-do Republic of Korea

**Keywords:** Esotropia, Strabismus, Surgery

## Abstract

**Purpose:**

To investigate the surgical results of the non-adjustable suture technique under general anesthesia for the correction of acute acquired comitant esotropia.

**Study design:**

Retrospective case study.

**Methods:**

Patients with acute acquired comitant esotropia who underwent corrective surgery from September 2008 to June 2018 were included. Surgical treatment was conducted based on the measured maximum angle after occlusion for at least 1 h; all surgeries were performed using the non-adjustable suture technique under general anesthesia. Motor success was categorized into three groups: good, ortho; fair, 2 to 8 prism diopters (PD); and poor, over 8 PD. Sensory success was divided into two groups: good (no diplopia with binocular vision) and poor (no stereopsis with diplopia).

**Results:**

40 patients (21 male and 19 female, 28.78 ± 15.32 years old) were included. Preoperative esodeviation was 28.0 ± 12.8 PD. Mean refractive error was − 2.5 ± 2.5 D (spherical equivalent). After the occlusion of one eye, 14 patients (35%) showed an esodeviation increase of more than 5 PD. There were 70% good, 25% fair, and 5% poor outcomes regarding motor success. 96% of the patients demonstrated good sensory success.

**Conclusion:**

The non-adjustable correction based on the maximum angle after 1 h had a relatively excellent motor and sensory success rate.

## Introduction

Acute acquired comitant esotropia (AACE) is an acute-onset esotropia with diplopia in older children and adults; notably, neurological examinations in these patients, including imaging tests, reveal negative results [1]. These features can distinguish AACE from other types of esotropia, such as infantile esotropia, accommodative esotropia, and paralytic strabismus.

Few reports of corrective surgical results have been published. Spierer reported that ten patients had good corrective surgical results [all patients were orthophoric or minimally esophoric and exhibited good stereopsis (40 s)] [2]. However, Sturm et al. revealed motor success within eight prism diopters (PD) in 92% of the patients; sensory success was present in only 60% [3]. Lee and Kim suggested that increasing the surgical dose is recommended to correct the sensory and motor success [4]. In order to increase the success rate, the adjustable suture technique is considerable. However, there is controversy regarding the success rates between adjustable and non-adjustable suture techniques [5]. Moreover, topical anesthesia has some side effects, including cardiac problems and pain [6, 7]. Therefore, we investigated the clinical features and surgical outcomes of the non-adjustable suture technique under general anesthesia in AACE patients.

## Methods

The study was a non-randomized, retrospective review of the medical records of 40 patients who were diagnosed with acute acquired comitant esotropia at Kim’s Eye Hospital between 2008 and 2016.

All patients showed comitant deviation in alternating gaze. We excluded patients under ten years old or a history of any of the following: ocular surgery, abnormal central nervous system findings, amblyopia, or head trauma. In order to focus on the Franceschetti type of AACE, AACE that developed after occlusion and orthotropia at near distance was excluded. The study was approved by the Institutional Review Board of Kim’s Eye Hospital and was conducted in accordance with the tenets of the Declaration of Helsinki. The IRB board of Kim’s Eye Hospital waived the requirement to obtain informed consent.

Full ophthalmologic examinations were performed, including slit-lamp examination, cycloplegic refraction, and alternate cover test. One eye with higher refractive errors was chosen in each patient, in order to investigate the relationship between refractive errors and esodeviation. All surgeries were performed under general anesthesia by one surgeon. The non-adjustable suture technique was used.

In order to determine the angle of surgery, the maximum deviation was measured after 1 h of occlusion. Preoperative and postoperative horizontal deviations were measured from a distance by using the alternating prism cover test with correction. Surgical success was divided into the motor and sensory successes. Motor and sensory successes were defined as shown in Table 1. The deviation was measured at a far distance. Sensory testing was performed by the Lang I stereotest (Lang-Stereotest AG, Küsnacht, Switzerland); the stereopsis result was positive when the patients could distinguish all three figures.

**Table 1:** Definition of surgical success

	Motor success	Sensory success
Good	Ortho	Stereopsis test: positive
Fair	2–8 PD*	-
Poor	over 8 PD	Stereopsis test: negative

*PD: prism diopters

Data were analyzed using SPSS 18.0 for Windows (SPSS Inc., Chicago, IL, USA). The Spearman correlation test was evaluated the relationship between refractive errors and postoperative deviation.

## Results

A total of 40 patients (21 male and 19 female) were included [mean age, 28.8 years (12–68 years)). Thirty-three of 40 underwent bilateral medial rectus muscle recession (BMR), and unilateral lateral rectus muscle resection and medial rectus muscle recession (RNR) were performed in seven patients. The surgical dose was performed according to Parks’ table.

**Fig. 1:**
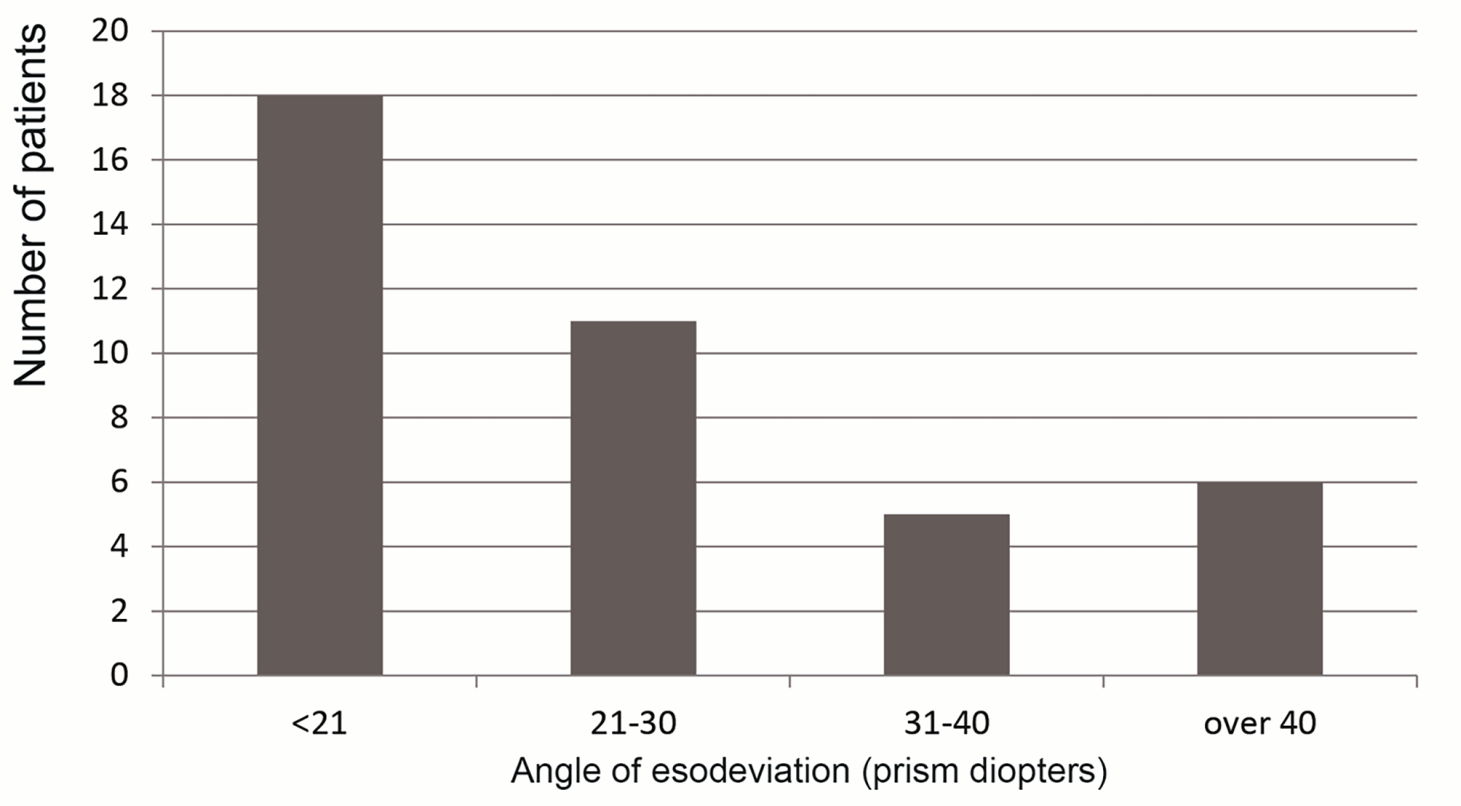
Preoperative esodeviation, measured after 1-hour occlusion

Preoperative esodeviation was 28.0 ± 12.8 prism diopters (PD) (Fig. 1); esodeviation increased more than 5 PD after 1-hour occlusion in 14 of 40 patients (35%). The increase in esodeviation after occlusion was an average of 9.5 PD (range, 5–35 PD).

**Fig. 2:**
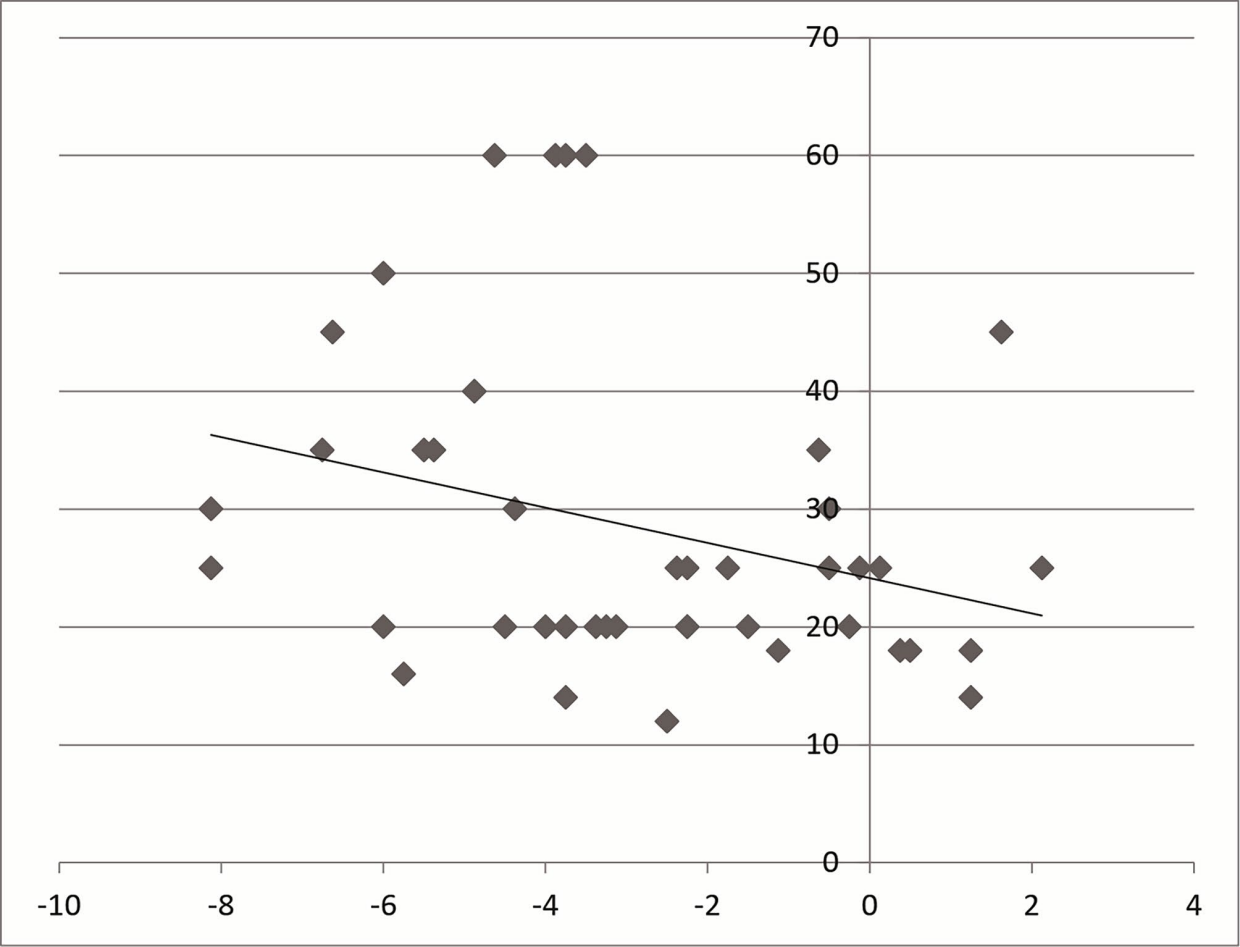
Correlation between refractive errors and esodeviation. More myopic patients tended to have greater esodeviation (*r* = 0.297, p = 0.048). (X-axis : refractive errors, Y-axis : angle of esodeviation)

The mean refractive error was − 2.5 D ± 2.5 D (spherical equivalent). There were seven hyperopic patients, 27 mild myopic patients (less than − 5 diopters), and six highly myopic patients. Patients with higher myopia tend to exhibit greater esodeviation than both less myopic and hyperopic patients (*r* = 0.297, p = 0.026) (Fig. 2).

**Fig. 3:**
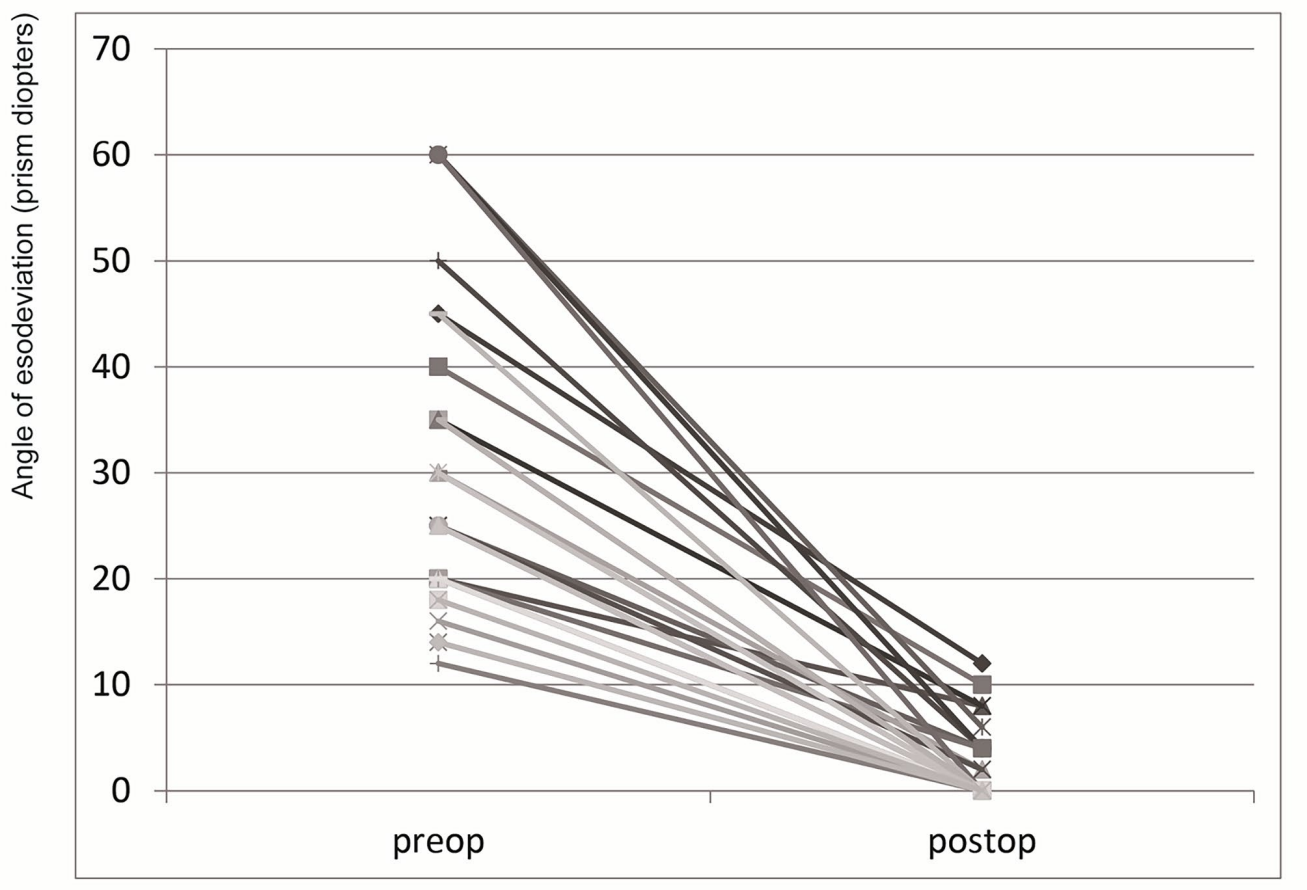
Surgical results of acute acquired comitant esotropia

The mean follow-up period was 17.2 ± 3.4 months. Motor success was obtained in all 40 patients; we could investigate sensory success in 28. 95% of the patients showed more than fair motor success. In the all fail motor groups, two out of 40 patients underwent bilateral medial rectus recession. After surgery, the greatest esodeviation was 12 PD; however, this patient showed normal stereopsis (Fig. 3). 93% of the patients had good stereopsis after surgery, and two patients who had no stereopsis showed 10 PD esodeviation (Table 2).

**Table 2:** Surgical outcomes of acute acquired comitant esotropia under general anesthesia

	Motor success	Sensory success
Good	28 (70%)	26 (93%)
Fair	10 (25%)	
Poor	2 (5%)	2 (7%)

## Discussion

This study showed that the results of non-adjustable surgery under general anesthesia for AACE are favorable in both motor and sensory aspects.

AACE can be categorized into three types: [1, 8] (1) Swan type, which develops after occlusion; (2) Franceschetti type, which exhibits a minimal amount of hypermetropia without underlying causes; (3) Bielschowsky type, which is related to uncorrected myopia of -5 D or greater. The present study excluded AACE that developed after occlusion or orthotropia at near distance. Recently, a new classification for AACE has been implemented, in which seven types of AACE are suggested (1) acute accommodative, (2) decompensated monofixation syndrome, (3) idiopathic, (4) the intracranial disease, (5) occlusion-related, (6) different etiology, and (7) cyclic type) [9]. Initially, we excluded patients with intracranial disease, different etiology, occlusion-related, and cyclic AACE. Our results are somewhat ambiguous in that classification depended on the three classical types of AACE, but the present patients had the Franceschetti type of AACE. However, most patients could be categorized as idiopathic.

More myopic patients showed greater esodeviation, which could be due to excessive near distance requirement in the myopia group, according to Bielschowsky’s theory [10, 11]. Myopia patients were the most common group in a Chinese AACE study [12], and the present study showed a similar result. AACE in adults can be associated with myopia [2]. However, this myopic preponderance may be epidemiological bias [13]. In cases of AACE related to hyperopia, full correction with glasses would help restore binocularity [14]. Esodeviation was not corrected with glasses in any of the present cases. Lee and Kim also mentioned that refractive errors had no significant relation to success rate [4].

Surgical studies regarding AACE have revealed favorable surgical results [2, 14, 15]. In order to improve surgical results, variable methods such as increasing surgical dose, preoperative prism adaptation, consideration of surgical methods, and adjustable surgery could be considerate [4, 15, 16]. Williams and Hoyt reported that stereopsis could be achieved after surgical treatment of AACE without neurologic abnormalities [17]. However, the previous study involved surgery with the adjustable technique. Our surgical results under general anesthesia revealed a favorable outcome and targeting the maximum angle of esodeviation after occlusion is a pivotal factor in improving the success rate. In the present study, failure of motor success was not found in unilateral RNR patients. Even though unilateral RNR has a more favorable motor outcome, the number of patients seems to be insufficient to analyze the effect of surgical methods.

This study has some limitations. We could not directly compare topical anesthesia, adjustable suture technique, and general anesthesia. Although topical anesthesia has advantages, such as surgical time, cost, and superior surgical results [18, 19], it could allow the sensation of pain during surgery, and intraoperative bradycardia, tachycardia, and asystole by oculocardiac reflex [7, 20]. The adjustable technique may provide better surgical outcomes based on the refined realignment of ocular muscles; however, there was no evidence of improved surgical outcomes in patients undergoing adjustable surgery [5, 21]. In addition, this study had no control group. Therefore, it was impossible to study whether this method was better or worse than the other methods such as adjustable surgery or techniques using other measurements of strabismus angle.

## Conclusion

A surgical correction based on the maximum angle after 1 h of occlusion showed a favorable motor and sensory success rate. Therefore, the non-adjustable suture technique under general anesthesia could be considered in AACE patients to avoid the side effects of topical anesthesia and the adjustable technique.

## Data Availability

The data used to support the findings of this study are available from the corresponding author upon request.
